# In vivo and in vitro characterization of GL0034, a novel long‐acting glucagon‐like peptide‐1 receptor agonist

**DOI:** 10.1111/dom.14794

**Published:** 2022-07-18

**Authors:** Ben Jones, Vinod Burade, Elina Akalestou, Yusman Manchanda, Zenouska Ramchunder, Gaëlle Carrat, Marie‐Sophie Nguyen‐Tu, Piero Marchetti, Lorenzo Piemonti, Isabelle Leclerc, Rajamannar Thennati, Tina Vilsboll, Bernard Thorens, Alejandra Tomas, Guy A. Rutter

**Affiliations:** ^1^ Section of Endocrinology and Investigative Medicine, Department of Metabolism, Digestion and Reproduction, Faculty of Medicine Imperial College London London UK; ^2^ High Impact Innovations—Sustainable Health Solutions Sun Pharmaceutical Industries Limited Vadodara India; ^3^ Section of Cell Biology and Functional Genomics, Department of Metabolism, Digestion and Reproduction, Faculty of Medicine Imperial College London London UK; ^4^ Department of Clinical and Experimental Medicine, Islet Cell Laboratory University of Pisa Pisa Italy; ^5^ Diabetes Research Institute IRCCS Ospedale San Raffaele Milan Italy; ^6^ CRCHUM University of Montréal Montréal Canada; ^7^ Clinical Metabolic Physiology, Steno Diabetes Center Copenhagen, Gentofte Hospital University of Copenhagen Copenhagen Denmark; ^8^ Center for Integrative Genomics University of Lausanne Lausanne Switzerland; ^9^ Lee Kong Chian School of Medicine Nanyang Technological University Singapore Singapore

**Keywords:** antidiabetic drug, antiobesity drug, beta‐cell function, drug development, GLP‐1 analogue, incretin therapy

## Abstract

**Aims:**

To describe the in vitro characteristics and antidiabetic in vivo efficacy of the novel glucagon‐like peptide‐1 receptor agonist (GLP‐1RA) GL0034.

**Materials and Methods:**

Glucagon‐like peptide‐1 receptor (GLP‐1R) kinetic binding parameters, cyclic adenosine monophosphate (cAMP) signalling, endocytosis and recycling were measured using HEK293 and INS‐1832/3 cells expressing human GLP‐1R. Insulin secretion was measured in vitro using INS‐1832/3 cells, mouse islets and human islets. Chronic administration studies to evaluate weight loss and glycaemic effects were performed in *db/db* and diet‐induced obese mice.

**Results:**

Compared to the leading GLP‐1RA semaglutide, GL0034 showed increased binding affinity and potency‐driven bias in favour of cAMP over GLP‐1R endocytosis and β‐arrestin‐2 recruitment. Insulin secretory responses were similar for both ligands. GL0034 (6 nmol/kg) led to at least as much weight loss and lowering of blood glucose as did semaglutide at a higher dose (14 nmol/kg).

**Conclusions:**

GL0034 is a G protein‐biased agonist that shows powerful antidiabetic effects in mice, and may serve as a promising new GLP‐1RA for obese patients with type 2 diabetes.

## 1 INTRODUCTION

Glucagon‐like peptide‐1 receptor agonists (GLP‐1RAs) provide substantial reductions in glycated haemoglobin (HbA1c) and significant body weight loss in type 2 diabetes (T2D) and obesity.^1^ GLP‐1RAs, with or without metformin based on glycaemic needs, are appropriate initial therapy for individuals with T2D with or at high risk of atherosclerotic cardiovascular disease and/or chronic kidney disease.^2^ Six glucagon‐like peptide‐1 receptor (GLP‐1R) mono‐agonists are approved by the US Food and Drug Administration for the treatment of patients with T2D, including short‐acting, intermediate‐acting and long‐acting therapies.^3^ Circulatory half‐life protraction of the latest generation of GLP‐1RAs is achieved by fatty acid conjugation and resultant binding to albumin (semaglutide), or by covalent linkage to Fc fragment of human IgG4 (dulaglutide), both of which increase effective molecular size sufficiently to virtually abolish clearance by renal filtration.^1^

Glucose lowering by GLP‐1RAs is attributable to a combination of effects, including a direct action on pancreatic beta‐cell GLP‐1Rs that potentiates glucose‐stimulated insulin secretion, suppression of glucagon release, delayed gastric emptying, insulin sensitization due to weight loss, and neuronally mediated reductions in hepatic glucose output.^4^ GLP‐1RA treatment also exerts further beneficial metabolic effects such as improvements in lipid profile and resolution of hepatic steatosis. Cardiovascular events and all‐cause mortality are also lowered in T2D.^5^

As a Gα_s_‐favouring G protein‐coupled receptor (GPCR), many cellular actions of GLP‐1R activation are attributable to elevations in intracellular cyclic adenosine monophosphate (cAMP).^6^ Recent studies have, however, highlighted the complexities of GLP‐1R signalling, including a possible role for Gα_q_, acting to mobilize intracellular Ca^2+^ ^7^ as well as modulation of GLP‐1R signal duration by β‐arrestin‐mediated desensitization and β‐arrestin‐independent endocytosis.^8^, ^9^ Ligand‐specific preference for distinct GLP‐1R‐coupled cellular signalling and trafficking responses, or biased agonism, is a potential means to extend the duration of GLP‐1RA action, typically through avoidance of receptor desensitization and downregulation.^10^

In this paper, we describe the properties of GL0034, a novel long‐acting, human GLP‐1RA based on the sequence of native GLP‐1. This agonist shares significant structural homology with the class‐leading GLP‐1RA semaglutide (Ozempic™) but with a distinct linker sequence between its polypeptide chain and albumin‐binding acyl group, which could potentially affect its pharmacological properties. We evaluate the performance of GL0034 in vitro and in vivo in comparison to other marketed compounds using a variety of models, including cell lines, primary pancreatic islets, *db/db* mice and diet‐induced obese mice, specifically aiming to identify evidence of biased agonism as a potential explanation for differences in antidiabetic efficacy.

## 2 MATERIALS AND METHODS

### 2.1 Study approvals

All in vivo studies performed at Imperial College London were approved by the College's Animal Welfare and Ethical Review Body according to the UK Home Office Animals Scientific Procedures Act, 1986 (Project Licenses PA03F7F0F to Isabelle Leclerc, and PD75F462C to Professor Kevin Murphy). All in vivo procedures at Sun Pharmaceuticals Inc. were approved by the Institutional Animal Ethics Committee (IAEC #608 and 650; V.B.). Studies involving human islets were approved by the National Research Ethics Committee (NRES) London (Fulham), Research Ethics Committee no. 07/H0711/114, and by relevant national and local ethics permissions, including consent from next of kin where required, at the isolation centres involved (see Table S1).

### 2.2 Peptide synthesis

GL0034 was synthesized using solid phase technology employing Fmoc amino acids and with suitable protections of other functional groups for coupling to grow the desired sequence. A further Lys residue was acylated, with deprotection at appropriate stages and purification on preparative high‐performance liquid chromatography, resulting in isolation of GL0034. Analysis of GL0034 by ultra‐high‐performance liquid chromatography‐high‐resolution mass spectrometry revealed that the obtained monoisotopic intact mass of the principal chromatography peak of GL0034 (4164.178) matches the theoretical monoisotopic mass (4164.1775). Chromatographic purity of the batches synthesized was approximately 97%.

### 2.3 Cell culture

HEK293 cells stably expressing human SNAP‐GLP‐1R (“HEK293‐SNAP‐GLP‐1R”) were described in an earlier publication^8^ and maintained in DMEM with 10% fetal bovine serum (FBS), 1% penicillin/streptomycin and G418 (1 mg/mL). Wild‐type INS‐1832/3 cells,^11^ or INS‐1832/3 cells lacking endogenous GLP‐1R after CRISPR/Cas9 deletion^12^ (a gift from Dr Jacqueline Naylor, Astra Zeneca) but stably expressing human SNAP‐GLP‐1R,^8^ were maintained in RPMI‐1640 with 11 mM glucose, 10 mM HEPES, 2 mM glutamine, 1 mM sodium pyruvate, 50 μM β‐mercaptoethanol, 10% FBS and 1% penicillin/streptomycin. PathHunter CHO‐K1‐βarr2‐EA‐GLP‐1R cells (DiscoverX) were maintained in F12 medium with 10% FBS, 1% penicillin/streptomycin, G418 (1 mg/mL) and hygromycin (250 μg/mL).

### 2.4 Time‐resolved FRET surface receptor binding assays

HEK293‐SNAP‐GLP‐1R cells were labelled in suspension with SNAP‐Lumi4‐Tb (40 nM, Cisbio, Codelet, France) for 1 hour at room temperature in complete medium. After washing and resuspension in hanks' balanced salt solution containing 0.1% bovine serum albumin and metabolic inhibitors (20 mmol/L 2‐deoxygucose and 10 mmol/L NaN_3_) to prevent GLP‐1R internalization,^13^ binding experiments were performed by time‐resolved förster resonance energy transfer (FRET) using exendin (9‐39) with fluorescein isothiocyanate (FITC) installed at position K12 as previously described.^14^ For further details, see Supplementary Methods.

### 2.5 cAMP assay

HEK293‐SNAP‐GLP‐1R cells were stimulated with agonist for 30 minutes at 37°C, either in serum‐free media (SFM) or SFM supplemented with 2% human serum albumin (HSA; Sigma Aldrich, Poole, UK). cAMP was determined at the end of the incubation period by homogenous time‐resolved fluorescence (HTRF; cAMP Dynamic 2 kit; Cisbio) using a Spectramax i3x plate reader (Molecular Devices, Wokingham, UK).

### 2.6 High content imaging endocytosis assay

The assay was performed as previously described.^15^ For further details see Supplementary Methods.

### 2.7 β‐Arrestin‐2 recruitment assay

PathHunter CHO‐K1‐βarr2‐EA‐GLP‐1R cells were stimulated with agonist for 30 minutes at 37°C in Ham's F12 medium without FBS. At the end of the incubation period, cells were lysed by addition of PathHunter detection reagents and luminescent signal was subsequently recorded from each well.

### 2.8 GLP‐1R internalization assay by confocal microscopy

INS‐1‐SNAP‐GLP‐1R cells were seeded the day before the assay into glass‐bottom dishes (MatTek Life Sciences, Ashland, Massachusetts). Adherent cells were then labelled with SNAP‐Surface® 549 (New England Biolabs UK, Hitchin, UK), washed with 1× phosphate‐buffered saline (PBS) and imaged in Live Cell Imaging Solution (Thermo Fisher, Waltham, Massachusetts). Time‐lapse confocal imaging was performed using a spinning disk confocal microscope with a 60× oil immersion objective. After a 1‐minute baseline recording, agonist was added to cells and the imaging continued for 10 minutes. Agonist‐mediated internalization was quantified in Fiji v1.53c as loss of membrane signal over time, after normalization to baseline membrane signal.

### 2.9 GLP‐1R recycling assay

INS‐1‐SNAP‐GLP‐1R cells were seeded the day before the assay in 96‐well imaging plates. Cells were washed twice with PBS, then stimulated with 100 nM of the respective agonist in complete media, with an equal number of wells kept as vehicles. Following stimulation of the cells for 1 hour, they were washed twice with PBS and labelled with exendin‐4‐TMR^14^ for either 1 or 3 hours, washed again with PBS, and imaged via high content microscopy as in Section 2.6.

### 2.10 Isolation and culture of mouse pancreatic islets

Male C57BL6/J mice were purchased from Charles River (Edinburgh, UK). The mice were housed in a pathogen‐free facility with a 12‐hour light/12‐hour dark cycle, with free access to standard mouse chow diet (RM‐1; Special Diet Services, Witham, UK) and water at the Imperial College Central Biomedical Services facility. Mice were killed by cervical dislocation and pancreatic islets isolated by collagenase digestion as previously described^16^ and cultured in RPMI 1640 medium, 11 mM glucose, supplemented with 10% (v/v) fetal bovine serum plus penicillin (100 units/mL), and streptomycin (0.1 mg/mL) at 37°C in an atmosphere of humidified air (95%) and CO_2_ (5%).

### 2.11 Human islet culture

Human islets were isolated from deceased heart‐beating donors and cultured in RPMI‐1640 (11879‐020) supplemented with 5.5 mM glucose, 10% FBS, 1% penicillin/streptomycin and 0.25 μg/mL fungizone. The characteristics of the donors and isolation centres are given in Table S1.

### 2.12 In vitro insulin secretion assays

Mouse islets (10/well) were incubated in triplicate for each condition and treatment. Islets were preincubated for 1 hour in 3 mM glucose Krebs‐Ringer HEPES bicarbonate buffer prior to secretion assay (30 minutes) in 3 mM or 11 mM glucose with vehicle, GL0034 or semaglutide. The secretion medium was then collected to measure the insulin and proinsulin concentrations using an insulin HTRF kit (Cisbio). INS‐1832/3 cells were treated with the indicated concentrations of GL0034 or semaglutide for 16 hours in complete RPMI‐1640 medium at 11 mM glucose. A sample of supernatant was collected and analysed for insulin content by HTRF.

### 2.13 Pharmacokinetic study

A plasma pharmacokinetic study of GL0034 was performed in male CD‐1 Mice (6‐9 weeks of age; 30‐50 g body weight) procured from the Laboratory Animal Resources Department of Sun Pharma Advanced Research Company Ltd (www.sparc.life, a subsidiary of Sun Pharma Industries Ltd.). For full details see Supplementary Methods.

### 2.14 Chronic administration study in *db/db* mice

*db*/*db* (C57BL/KsJ‐db/db) male/female mice (age 7‐11 weeks; body weight 35‐60 g), procured from the Laboratory Animal Resources Department of Sun Pharma Advanced Research Company Ltd, were housed in individual ventilated cages with free access to food and water and maintained on a 12‐hour light/12‐hour dark cycle. The mice were acclimatized for 3 days. On Day 0, each animal was weighed using a digital weighing balance and 10 μL blood was collected by retro‐orbital plexus puncture under mild isoflurane anaesthesia. Blood glucose level was measured using a hand‐held blood glucose meter (One Touch™ Ultra™; LIFESCAN, Johnson & Johnson, Malvern, Pennsylvania) and % HbA1c was measured using A1CNow + ® kits (PTS Diagnostics, Indianapolis, Indiana). The mice were divided into treatment groups (n = 8 per group; four male, four female) with matched baseline HbA1c levels (mean 56 mmol/mol). Vehicle, GL0034 (1.5, 3 and 6 nmol/kg), semaglutide (14 nmol/kg) or dulaglutide (1.5 nmol/kg) was injected subcutaneously into the neck region of the mice on every other day for 4 weeks. Body weight and food intake were monitored serially. On Day 29 of the study, 24 hours after the last dose, blood was collected for measurement of HbA1c, triglycerides (Triglyceride Colorimetric Assay Kit, Item No. 10010303; Cayman Chemicals, Ann Arbor, Michigan), insulin (Cat. No. EZRMI‐13 K; Merck, Rahway, New Jersey), C‐peptide (Mouse C‐Peptide ELISA, Cat. No. 80‐CPTMS‐E01; ALPCO, Salem, New Hampshire) and glucagon (Glucagon ELISA, Cat no. 48‐GLUHU‐E01; ALPCO). Beta‐cell function was assessed using homeostatic model assessment index (HOMA‐B)^17^ from glucose and insulin concentrations.

### 2.15 Chronic administration study in high‐fat, high‐sucrose diet‐fed mice

Male C57BL6/J mice were purchased from Charles River (UK). The mice were housed in a pathogen‐free facility with a 12‐hour light/12‐hour dark cycle. From the age of 8 weeks they were put on a 58 kcal % fat and sucrose diet (D12331; Research Diets Inc., New Brunswick, New Jersey) for 8 weeks to induce obesity and glucose intolerance. The mice were then injected subcutaneously every 48 hours with saline, semaglutide (14 nmol/kg) or GL0034 (6 nmol/kg) for 4 weeks. Body weight was monitored at regular intervals. For glucose tolerance testing, mice were fasted overnight (total 16 hours) and given free access to water. At 9:00 am, glucose (3 g/kg body weight) was administered via intraperitoneal injection or oral gavage. Blood was sampled from the tail vein at 0, 5, 15, 30, 60 and 90 minutes after glucose administration. Blood glucose was measured with an automatic glucometer (Accuchek; Roche, Burgess Hill, UK). To quantify circulating insulin, 10 μL blood was collected from the tail vein into heparin‐coated tubes (Sarstedt, Beaumont Leys, UK). Plasma was separated by sedimentation at 10 000*g* for 10 minutes at 4°C. Plasma insulin levels were measured in 5‐μL aliquots by ELISA kits from Crystal Chem (Elk Grove Village, Illinois). At the end of the study, pancreata were isolated and processed for immunohistochemistry.

### 2.16 Immunohistochemistry of pancreas sections

Isolated pancreata were fixed in 10% (vol/vol) buffered formalin and embedded in paraffin wax within 24 hours of removal. Slides (5 μm) were submerged sequentially in Histoclear (Sigma‐Aldrich) followed by washing in decreasing concentrations of ethanol to remove paraffin wax. Permeabilized pancreatic slices were blotted with ready‐diluted anti‐guinea pig insulin (Agilent Technologies, Santa Clara, California) and anti‐mouse glucagon (Sigma‐Aldrich) primary antibody (1:1000). Slides were visualized by subsequent incubation with Alexa Fluor 488‐ (1:1000) and 568‐labelled donkey anti‐guinea pig and anti‐mouse antibody (1:1000). Samples were mounted on glass slides using Vectashield™ (Vector Laboratories, Newark, California) containing DAPI. Images were captured on a Zeiss Axio Observer.Z1 motorized inverted widefield microscope, fitted with a Hamamatsu Flash 4.0 Camera, using a Plan‐Apochromat 206/0.8 M27 air objective with Colibri.2 LED illumination. Data acquisition was controlled with Zeiss Zen Blue 2012 Software. Fluorescence quantification was achieved using Image J (https://imagej.nih.gov/ij/). Whole pancreas was used to quantitate cell mass.

### 2.17 Statistical analysis

For in vitro data, the average of within‐assay replicates was counted as one biological replicate. For kinetic binding assays, rate constants for association and dissociation of the unlabelled ligands were calculated using the “kinetics of competition binding” algorithm in prism 8 (GraphPad Software), using values for exendin(9‐39)‐FITC determined in the same experiment. Functional concentration response data were fitted using three‐parameter logistic fitting (prism 8), with constraints applied where appropriate. Pharmacokinetic analysis was performed using Phoenix® Win‐Nonlin® (Version 8.0). Statistical comparisons were performed using Student's t‐test or one‐way ANOVA, as appropriate. Paired or matched analyses were performed where permitted by the experimental design. Specific post hoc tests are indicated in the legends. Statistical significance was assigned where *P* < 0.05. Data are represented as mean ± standard error of the mean (SEM) throughout, with individual replicates indicated where possible.

## 3 RESULTS

### 3.1 GL0034 shows increased G protein‐biased agonism compared to semaglutide

**FIGURE 1:**
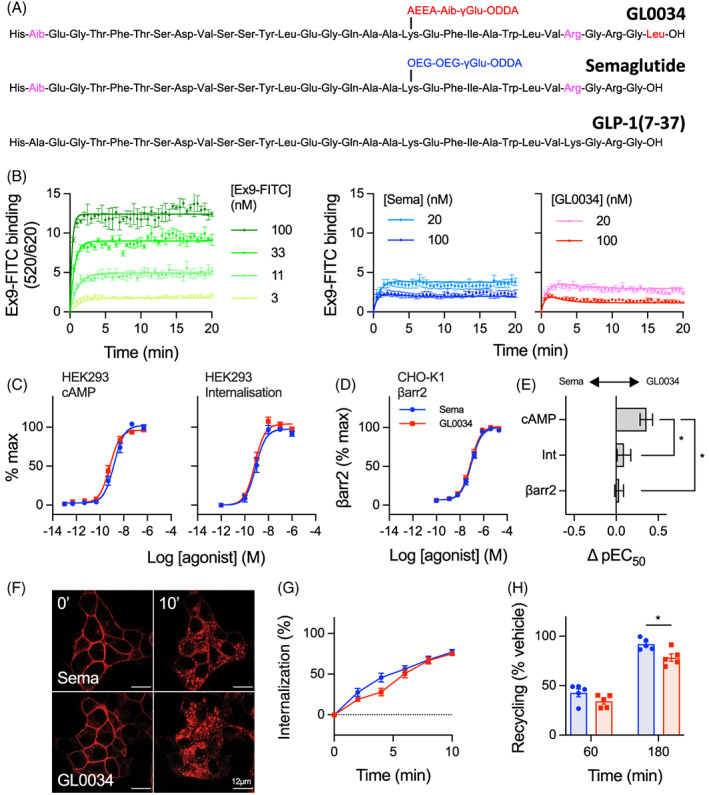
Pharmacological characterization of GL0034 in comparison to semaglutide. (A) Amino acid sequences of glucagon‐like peptide‐1 [GLP‐1](7‐37), semaglutide and GL0034. (B) Binding of exendin(9‐39)‐ fluorescein isothiocyanate (Ex9‐FITC) to HEK293‐SNAP‐ glucagon‐like peptide‐1 receptor (GLP‐1R) cells with and without coaddition of indicated concentration of GL0034 or semaglutide (Sema), n = 4. (C) cAMP production and GLP‐1R internalization measured in parallel HEK293‐SNAP‐GLP‐1R cells, with three‐parameter logistic curve fitting of pooled data from n = 5 repeats. (D) β‐arrestin‐2 recruitment in PathHunter‐GLP‐1R‐EA‐βarr2 cells, n = 5, (E) subtracted pEC_50_ estimates from (C) and (D), compared by one‐way ANOVA with Tukey's test. (F) Representative images showing GLP‐1R internalization in INS‐1‐SNAP‐GLP‐1R cells stimulated by 100 nM agonist. (G) Quantification of internalized GLP‐1R from n = 4 repeats of experiment shown in (F). (H) Quantification of exendin‐4‐TMR uptake by INS‐1‐SNAP‐GLP‐1R cells after prior treatment with 100 nM semaglutide or GL0034, indicating re‐emergence of internalized GLP‐1R over time, from n = 5 experiments and comparison by two‐way repeated‐measures ANOVA with Sidak's test. *P < 0.05. Data represented as mean ± SEM

The structure of GL0034 is shown in Figure 1A alongside that of native GLP‐1(7‐37) and the current class‐leading GLP‐1RA, semaglutide. Like semaglutide, GL0034 differs from GLP‐1 by the substitution of the native second position amino acid alanine with 2‐aminoisobutyric acid (Aib), which confers resistance to cleavage by dipeptidyl‐dipeptidase 4 (DPP‐4). Both GL0034 and semaglutide also feature a lysine→arginine substitution at position 28, which allows site‐specific acylation at the remaining lysine at position 20. GL0034 also has an additional C‐terminal leucine, not found in semaglutide, and a distinct linker sequence that couples the peptide to an octadecanedioic acid albumin‐binding moeity. The C‐terminal leucine was included after an internal screening programme revealed beneficial effects of this modification on antidiabetic and weight loss efficacy in *db/db* mice (Table S2). The linker fragment includes Aib to provide stability towards peptidases and to balance hydrogen bonding donor and acceptor properties in the whole side chain with acyl group.

To determine how the above structural differences between GL0034 and semaglutide might affect ligand pharmacology, we first assessed the binding kinetic parameters of each agonist using a competition‐based assay in which binding of the fluorescent antagonist exendin(9‐39)‐FITC to GLP‐1R is monitored in real time in the presence of varying concentrations of test agonists.^10^ GL0034 showed a moderate slowing in dissociation kinetics compared to semaglutide, translating to a greater than doubling in binding affinity (Figure 1B, Table 1). cAMP accumulation and GLP‐1R endocytosis measured using the same cell model, the latter using a high content imaging‐based assay, confirmed that signalling potency was increased without a corresponding increase in internalization (Figure 1C, Table 1). We also measured β‐arrestin‐2 recruitment responses with both ligands (Figure 1D, Table 1), which showed no differences in response potency or E_max_. Comparison of potency differences within each assay confirmed significant signalling selectivity in favour of cAMP for GL0034 compared to endocytosis and β‐arrestin‐2 recruitment (Figure 1E).

**TABLE 1:** Pharmacological characteristics of GL0034 compared to semaglutide

Assay and cell model	Unit of measurement	Semaglutide	GL0034
Kinetic binding (HEK293‐SNAP‐GLP‐1R; Figure 1B)	K_on_ (M^‐1^, min^−1^)	8.0 × 10^6^ ± 1.0 × 10^6^	9.5 × 10^6^ ± 1.2 × 10^6^
	K_off_ (min^‐1^)	0.41 ± 0.02	0.22 ± 0.02 *
	Log K_d_ (M)	−7.3 ± 0.0	−7.6 ± 0.0 *
cAMP (HEK293‐SNAP‐GLP‐1R; Figure 1C)	LogEC_50_ (M)	−8.8 ± 0.2	−9.2 ± 0.1 *
	E_max_ (%)	103 ± 3	97 ± 2
Endocytosis (HEK293‐SNAP‐GLP‐1R; Figure 1C)	LogEC_50_ (M)	−9.1 ± 0.2	−9.2 ± 0.1
	E_max_ (%)	98 ± 2	105 ± 2
βarr2 (CHO‐K1‐βarr2‐EA‐GLP‐1R; Figure 1D)	LogEC_50_ (M)	−7.0 ± 0.1	−7.1 ± 0.1
	E_max_ (%)	100 ± 1	102 ± 1
Insulin secretion (INS‐1832/3; Figure 2C)	LogEC_50_ (M)	−8.2 ± 0.1	−8.2 ± 0.1
	E_max_ (fold increase)	2.5 ± 0.2	2.6 ± 0.3

**FIGURE 2:**
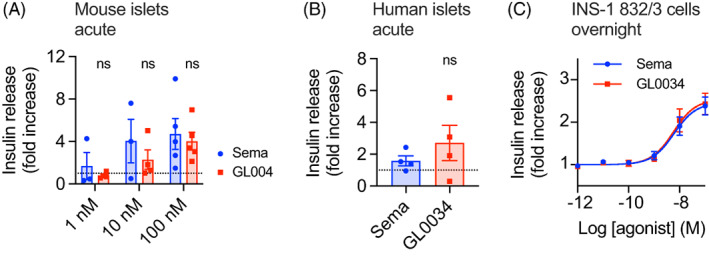
Comparable in vitro insulin secretory effects of GL0034 and semaglutide. (A) Insulin secretion from mouse islets stimulated for 30 minutes with 1 nM, 10 nM or 100 nM at 11 mM glucose, represented as fold increase versus 11 mM glucose alone, n = 3‐5, paired t‐tests. (B) As for (A) but with human islets, n = 4. (C) Insulin secretion from INS‐1832/3 beta cells stimulated at 11 mM glucose with a range of agonist concentrations for 16 hours, n = 5. Data represented as mean ± SEM, with individual replicates shown where possible

Data are estimates ± SEM from data shown in Figures 1 and 2. Statistical significance for GL0034 versus semaglutide, determined by paired t‐test, is indicated as * for *P* < 0.05.

To examine the GLP‐1R trafficking effects of each ligand in a beta‐cell context, we performed time‐lapse confocal microscopy of SNAP‐GLP‐1R‐expressing INS‐1832/3 cells (Figure 1F). Both ligands were able to induce rapid GLP‐1R endocytosis (Figure 1G), with no clear difference in kinetics observed between the two. GLP‐1R recycling was also determined by incubating cells with fluorescent exendin‐4‐TMR after an initial internalization step, with measured fluorescence uptake indicative of replenishment of plasma membrane GLP‐1R after semaglutide or GL0034 pretreatment and washout. This approach revealed modestly reduced recycling with GL0034, which is compatible with previous evidence showing that lower GLP‐1RA dissociation rates are associated with slower GLP‐1R recycling^10^ (Figure 1H).

Overall, these data reveal moderate increases in GLP‐1R binding affinity and cAMP signalling for GL0034 compared to semaglutide, without a corresponding increase in receptor endocytosis. Therefore, GL0034 shows G protein‐biased agonism relative to semaglutide.

### 3.2 Insulin secretory profiles in mouse and human islets

Static acute insulin secretion experiments using C57BL/6 mouse islets at 11 mM glucose demonstrated potentiation of glucose‐stimulated insulin secretion by both semaglutide and GL0034 at 10 and 100 nM, with no difference between the effect of either agonist (Figure 2A). Similarly, using islets from human donors, both semaglutide and GL0034 increased insulin secretion compared to 11 mM glucose alone, with a nonsignificant trend favouring GL0034 (Figure 2B). In case GL0034 and semaglutide differed in their ability to desensitize the GLP‐1R, we also measured insulin release after overnight incubation of INS‐1832/3 cells with a range of concentrations of each agonist; responses were virtually indistinguishable between the two compounds (Figure 2C, Table 1). These data demonstrate that, as expected, GL0034 is capable of potentiating insulin release at elevated glucose concentrations, but do not provide evidence of an increased insulinotropic effect of GL0034 compared to semaglutide.

### 3.3 Long‐lasting and potent antidiabetic effect of GL0034 in *db/db* mice

**FIGURE 3:**
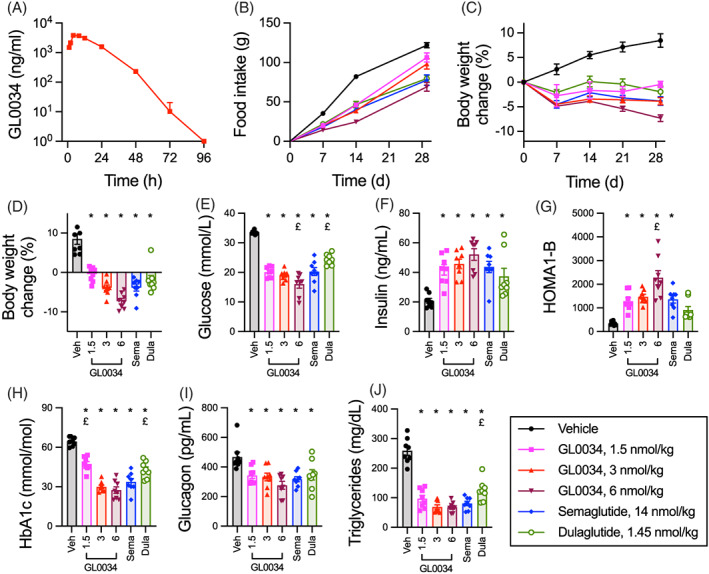
GL0034 has powerful antidiabetic effects in vivo. (A) Plasma GL0034 concentration following single subcutaneous injection (1 mg/kg) in male CD‐1 mice (n = 5 per timepoint). See also Table 2. (B) Cumulative food intake during a 28‐day study in *db/db* mice (n = 8/group, average body weight at study initiation = 48.3 ± 0.7 g) receiving alternative day subcutaneous injection of the indicated agonist. (C) As for (B) but showing body weight reduction. (D) Total body weight loss at the end of the study, with comparison by one‐way ANOVA with Holm Sidak's test to compare groups. (E) As for (D) but for plasma glucose concentration. (F) As for (D) but insulin concentration. (G) As for (D) but HOMA‐B as a measure of beta‐cell function. (H) As for (D) but % glycated haemoglobin (HbA1c). (I) As for (D) but plasma glucagon concentration. (J) As for (D) but plasma triglyceride concentration. *P < 0.05 versus vehicle, £ *P* < 0.05 versus semaglutide by indicated statistical test. Data represented as mean ± SEM, with individual replicates shown where possible. Dula, dulaglutide; Sema, semaglutide; Veh, vehicle

The pharmacokinetics of GL0034 were evaluated in CD‐1 mice (Figure 3A, Table 2). Plasma half‐life after subcutaneous injection was approximately 7.65 hours, which is comparable to semaglutide administered at the same dose.^18^ This was supported by comparisons of GLP‐1R cAMP signalling potency measured with and without 2% HSA, which indicated both ligands were extensively and similarly bound to albumin (∆pEC_50_ = 2.0 ± 0.2 [semaglutide] versus 2.1 ± 0.2 [GL0034]; *n* = 4, *P* > 0.05 by paired t‐test). We performed a chronic administration study in *db/db* mice with three separate dosing regimens of GL0034, as well as semaglutide and dulaglutide. Mice were injected subcutaneously every other day for 28 days with 1.5, 3 or 6 nmol/kg GL0034. Semaglutide was dosed at 14 nmol/kg every other day, providing dose equivalence to a maximally effective dose in mice.^19^ Dulaglutide, another commonly used once‐weekly injectable GLP‐1RA,^20^ was dosed every other day at 1.45 nmol/kg as an additional comparator. All ligand doses tested led to marked improvements in important metabolic readouts compared to vehicle control, including reduced energy intake, body weight reduction, glucose, insulin levels, and beta‐cell function as measured by HOMA‐B, HbA1c, and suppression of plasma glucagon and triglyceride levels (Figure 3B‐J). Notably, GL0034 at 3 nmol/kg matched the effects of semaglutide at >4 times higher dose, and at 6 nmol/kg, the effects of GL0034 were superior for some readouts, including weight loss (Figure 3C,D), glucose (Figure 3E) and HOMA‐B (Figure 3G). GL0034 also outperformed dulaglutide. GL0034 appears therefore to possess desirable antidiabetic characteristics.

**TABLE 2:** Pharmacokinetic analysis of GL0034

AUC_0‐t_, h.nmol/L	AUC_0‐inf_, h.nmol/L	C_max_, nmol/L	T_max_, h	t_1/2_, h	K_el_, 1/h
21 568.8	21 595.8	920.8	4	7.652	0.0906

Plasma pharmacokinetic parameter estimates following a single subcutaneous injection of GL0034 (1 mg/kg) in male CD‐1 mice (*n* = 5 per timepoint). See Figure 3A.

Abbreviations: AUC, area under the curve; C_max_, maximum concentration; T_1/2_, half time; Kel, elimination rate constant.

### 3.4 Effects of GL0034 in high‐fat, high‐sucrose diet‐fed mice

**FIGURE 4:**
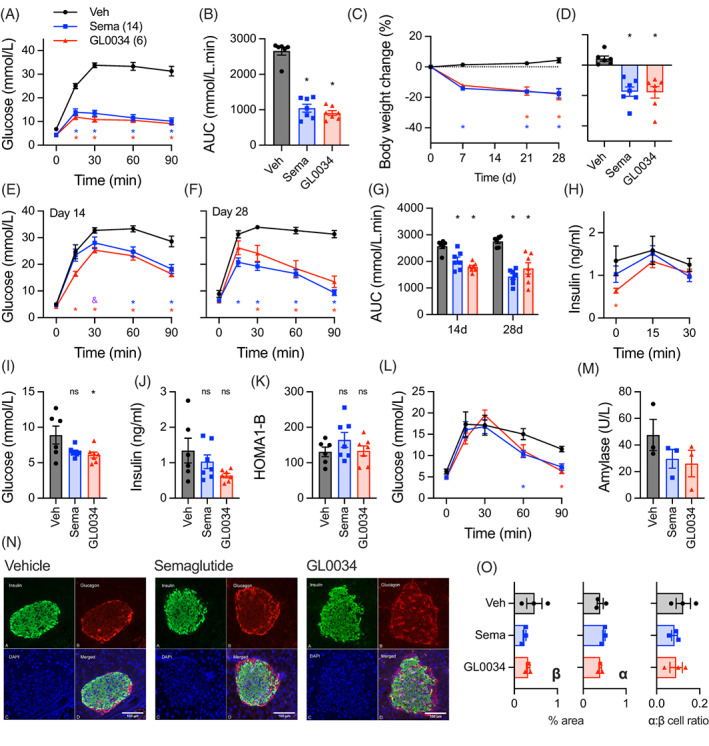
Effects of GL0034 and semaglutide in high‐fat, high‐sucrose‐fed (HFHS) mice. (A) Intraperitoneal glucose (3 g/kg) tolerance test (IPGTT) in HFHS mice 2 hours after subcutaneous administration of GL0034 (6 nmol/kg; n = 7), semaglutide (14 nmol/kg; n = 7) or vehicle (veh; n = 6), with two‐way repeated measures ANOVA and Tukey's test. (B) Area under the curve (AUC) analysis from (A), one‐way ANOVA with Tukey's test. (C) Body weight change over 4 weeks with subcutaneous administration of GL0034 (6 nmol/kg) or semaglutide (14 nmol/kg) every 48 hours, two‐way repeated‐measures ANOVA and Tukey's test. (D) Body weight loss at the end of the study, one‐way repeated‐measures ANOVA and Tukey's test. (E) IPGTT at 14 days, performed 48 hours after agonist dosing, with two‐way repeated measures ANOVA and Tukey's test. (F) As for (E) but at 28 days. (G) AUC analysis from (E) and (F), one‐way ANOVA with Tukey's test. (H) Plasma insulin concentrations from IPGTT shown in (F), two‐way repeated measures ANOVA and Tukey's test. (I) Fasting blood glucose from (F), one‐way ANOVA with Tukey's test. (J) Fasting plasma insulin from (H), one‐way ANOVA with Tukey's test. (K) HOMA1‐B calculated from (I) and (J), one‐way ANOVA with Tukey's test. (L) Oral glucose tolerance test (3 g/kg) performed at the end of the study, 48 hours after agonist dosing, with two‐way repeated‐measures ANOVA and Tukey's test. (M) Plasma amylase concentration from the end of the study, n = 3. (N) Representative immunohistochemical images from pancreata stained for insulin (green) or glucagon (red); scale bar = 100 μm. (O) Quantification of alpha (α) and beta (β)‐cell mass from (N), *n* = 3. * *P* < 0.05 versus vehicle (with colour coding: Blue = semaglutide, red = GL0034), £ *P* < 0.05 GL0034 versus semaglutide, by indicated statistical test. Data represented as mean ± SEM, with individual replicates shown where possible. Sema, semaglutide; Veh, vehicle

To corroborate our results from *db/db* mice, we performed a second study in mice fed a high‐fat, high‐sucrose (HFHS) diet to induce glucose intolerance. Acutely, GL0034 at 6 nmol/kg was at least as effective as semaglutide at 14 nmol/kg for lowering blood glucose in an intraperitoneal glucose tolerance test conducted 2 hours after agonist administration (Figure 4A,B). When injected every other day at the same doses, both agonists led to effective and sustained weight loss over 4 weeks (Figure 4C,D). Intraperitoneal glucose tolerance tests performed at 14 and 28 days, both at the end of the 48‐hour dosing window, showed similar effects of GL0034 and semaglutide on glucose tolerance (Figure 4E‐G) and insulin secretion (Figure 4H). Of note, fasting glucose from this study was somewhat lower with GL0034 than with semaglutide treatment (Figure 4I), in agreement with findings from *db/db* mice (Figure 3E). However, as fasting insulin was also lower (Figure 4J), there was no clear difference in HOMA‐B‐estimated measurement of beta‐cell function (Figure 4K). The effect of GL0034 on oral glucose tolerance at the end of the study was similar to that of semaglutide (Figure 4L). There was no difference in plasma amylase levels with either agonist (Figure 4M). We also found no clear evidence of agonist‐specific changes in beta‐ or alpha‐cell mass in pancreata harvested from a subset of mice from the study (Figure 4N,O).

## 4 DISCUSSION

We present here an in vitro and in vivo evaluation of GL0034, a novel long‐lasting GLP‐1RA with partial sequence homology to semaglutide. Despite being administered at a lower dose, GL0034 was at least as effective for glucose lowering and other metabolic effects, compared to semaglutide, in *db/db* and HFHS‐fed mice. These findings thus highlight the promise of this novel agent for the treatment of T2D. Of note, our findings seem likely to be linked to both higher binding affinity and G protein‐biased agonism with GL0034.

GL0034 shares substantial structural similarities with semaglutide, including a DPP‐4 resistance‐conferring alanine→2‐aminoisobutyric acid substitution at position 2, as well as an octadecanedioic acid group to promote reversible albumin binding. These similarities probably account for the very similar pharmacokinetic properties we observed with GL0034 compared to publicly available data for semaglutide.^18^ However, the linker strategy used to couple acyl moiety to peptide differs between GL0034 and semaglutide, and this may account for the in vitro pharmacological differences between the two agents, including increased GLP‐1R binding affinity with GL0034. As part of the development of semaglutide, it was shown that different linkers could have a major impact on GLP‐1R engagement, with cAMP signalling potency reduced by over 1000‐fold in some cases.^21^ We conclude that the novel linker sequence of GL0034 facilitates improvements in GLP‐1R binding without leading to an unacceptable reduction in pharmacokinetics.

We and others have previously demonstrated how G protein‐selective biased agonism is associated with profound alterations to GLP‐1R trafficking, resulting in improved insulinotropic and antidiabetic effects through avoidance of GLP‐1R desensitization and downregulation.^10^, ^14^, ^15^, ^22^, ^23^, ^24^, ^25^, ^26^, ^27^, ^28^ The majority of these examples showed “efficacy‐dominant” G protein bias, in which reductions in maximum obtainable GLP‐1R endocytosis or β‐arrestin recruitment appeared necessary to drive the improvements in sustained duration of action. Interestingly, most efficacy‐driven G protein‐biased agonists in this class also showed moderately reduced efficacy for coupling to G protein signalling, but signal amplification within the Gα_s_/cAMP/protein kinase A pathway allowed for full downstream responses, ultimately leading to paradoxically increased efficacy due to reduced desensitization. In contrast, another G protein‐biased GLP‐1R (“P5”) shows more modest reductions in transducer coupling efficacy, smaller changes in GLP‐1R desensitization and downregulation, and does not show enhanced insulinotropic efficacy in vitro or in vivo.^24^, ^29^ The cAMP‐favouring bias observed with GL0034 clearly resulted from increased cAMP potency compared to semaglutide, rather than via reduced efficacy for endocytosis or β‐arrestin‐2 recruitment. In keeping with the paradigm of efficacy‐driven bias being required for augmentation of insulin secretion, GL0034 showed no enhancement in sustained insulin secretion in vitro. As a further consideration, it is notable that GLP‐1R recycling with GL0034 was subtly delayed compared with semaglutide treatment, which would be expected to mitigate against any hypothetical increases in sustained insulin secretion by restricting the availability of surface GLP‐1Rs during continuous exposure. On the other hand, it is unclear whether drug concentrations achieved in vivo are sufficient to downregulate a significant proportion of surface GLP‐1Rs.

GL0034 showed potent antidiabetic effects in *db/db* and HFHS mice. Even at an almost 10‐fold lower dose (1.5 nmol/kg vs. 14 nmol/kg), blood glucose and insulin secretory improvements with 4 weeks of GL0034 treatment were equivalent to those seen with semaglutide in *db/db* mice. At the highest GL0034 dose tested (6 nmol/kg), still under half that of semaglutide, blood glucose lowering and HOMA‐estimated beta‐cell function were significantly better, with trends towards greater effects on HbA1c and suppression of plasma glucagon. GL0034 at 6 nmol/kg also matched metabolic effects of semaglutide at 14 nmol/kg in HFHS‐fed mice, including outperforming semaglutide for lowering fasting blood glucose. The potential to reduce manufacturing costs due to lower drug dosage is particularly appealing when one considers the drive towards oral GLP‐1RA formulations, which inevitably require considerably higher doses due to low bioavailability.^30^, ^31^ It is important to highlight that 6 nmol/kg GL0034 also led to greater body weight loss than semaglutide in *db/db* mice (although not in the HFHS study); this is, of course a therapeutic advantage, but also a clear confounder when wishing to understand differences in other metabolic parameters. Additional weight loss is a likely explanation for the enhanced effects of GL0034 that does not require agonist‐specific differences in direct action on the islet, particularly in the absence of any measured differences in acute or sustained insulin secretion between agonists in our in vitro studies. Notably, the middle dose of GL0034 tested (3 nmol/kg) showed very similar effects on body weight to semaglutide, and almost identical effects on other metabolic parameters.

Our study has several limitations. We performed most of our pharmacological evaluations using human GLP‐1R‐expressing systems, whereas it could be argued that a link to in vivo effects would be better represented by mouse GLP‐1R studies. On the other hand, orthosteric GLP‐1R‐biased agonist effects tend to be similar at rodent and human GLP‐1R,^25^, ^26^ and establishing the pharmacology in a human system is more translationally relevant. We focused in our study on Gα_s_‐dependent cAMP signalling on the basis that this is the primary Gα subtype coupled to GLP‐1R activation^32^, ^33^; however, a recent study has elevated the possibility of a role for Gα_q_‐dependent signalling,^7^ which is a subject for future investigation. We cannot exclude subtle differences in agonist pharmacokinetics, particularly during the chronic administration studies, as plasma drug levels were below the lower limit of quantification for both ligands at the sampling time. However, comparison of our single‐dose pharmacokinetic data for GL0034 with published values for semaglutide obtained using an apparently identical system, as well as cAMP signalling potency estimates with and without added albumin, argue against major differences. We also note that, whilst we evaluated insulin secretion in mouse islets and clonal INS‐1832/3 beta cells across a wide range of agonist concentrations, in human islets our data are limited to the effects on insulin secretion of high doses (likely exceeding those observed after injection in vivo) of the drug. Finally, our in vivo studies were primarily aimed at establishing the therapeutic effects of repeated GL0034 administration, meaning that we were unable to assess effects in the absence of weight loss. However, by using different GL0034 dosing regimens, we could compare GL0034 against comparator GLP‐1RAs at equivalent weight loss.

In conclusion, GL0034 is a novel GLP‐1RA showing potency‐driven G protein‐biased agonism with significant potential for the treatment of T2D.

## CONFLICTS OF INTEREST

Vinod Burade and Thennati Rajamannar are employees of Sun Pharmaceuticals, from whom Guy A. Rutter and Alejandra Tomas have received grant funding.

## Supporting information


**Appendix S1** Supporting Information

## Data Availability

Data available in article and in supplementary material.
