# Supplementary material for: In vivo and in vitro characterization of GL0034, a novel long‐acting glucagon‐like peptide‐1 receptor agonist
Source: Diabetes Obes Metab. 2022 Jul 18;24(11):2090–101. doi: 10.1111/dom.14794 (PMC9796023; doi:10.1111/dom.14794)
Supplement: Supplementary file 1 — Appendix S1 Supporting Information [file DOM-24-2090-s001.docx]

**Supplementary Information**

***In vivo* and *in vitro* characterisation of GL0034, a novel long-acting GLP-1 receptor agonist**

Ben Jones^1^, Vinod Burade^2^, Elina Akalestou^3^, Yusman Manchanda^3^, Zenouska Ramchunder^3^, Gaëlle Carrat^3^, Marie-Sophie Nguyen-Tu^3^, Piero Marchetti^4^, Lorenzo Piemonti^5^, Isabelle Leclerc^3^, Thennati Rajamannar^2^, Tina Vilsboll^6^, Bernard Thorens^7^, Alejandra Tomas^3,*^ and Guy A. Rutter^3,8,9,*^

^1^ Section of Endocrinology and Investigative Medicine, Department of Metabolism, Digestion and Reproduction, Faculty of Medicine, Imperial College London, U.K.

^2^ High Impact Innovations – Sustainable Health Solutions Unit, Sun Pharmaceutical Industries Limited, Vadodara, Gujarat, India

^3^ Section of Cell Biology and Functional Genomics, Department of Metabolism, Digestion and Reproduction, Faculty of Medicine, Imperial College London, U.K.

^4^ Department of Clinical and Experimental Medicine, Islet Cell Laboratory, University of Pisa, 56126, Pisa, Italy

^5^ Diabetes Research Institute, IRCCS Ospedale San Raffaele, Milano, Italy

^6^ Clinical Metabolic Physiology, Steno Diabetes Center Copenhagen, Gentofte Hospital, University of Copenhagen, Copenhagen, Denmark

^7^ University of Lausanne, Lausanne, Switzerland

^8^ Lee Kong Chian School of Medicine, Nanyang Technological University, 637553, Singapore

^9^ CRCHUM, University of Montréal, QC, Canada (CR-CHUM)

*Correspondence to Professor Guy Rutter, [g.rutter@imperial.ac.uk](mailto:g.rutter@imperial.ac.uk) or [guy.rutter.chum@ssss.gouv.qc.ca](mailto:guy.rutter.chum@ssss.gouv.qc.ca), or Dr Alejandra Tomas, a.tomas-catala@imperial.ac.uk

**Running title**: Anti-diabetic effects of GL0034

**Contents:**

- **Supplementary Methods.**
- **Supplementary Table 1.** Human islet preparations
- **Supplementary Table 2.** Internal screening of C-terminal Leu semaglutide analogue in db/db mice

**Supplementary Methods**

**TR-FRET binding assay**

The initial parts of the assay are described in the main methods. After a baseline read, TR-FRET signals were measured at regular intervals before and after addition of different concentrations of exendin(9-39)-FITC, or different concentrations of unlabelled agonist in combination with a fixed concentration (10 nM) of exendin(9-39)-FITC at 37°C. Measurements were made using a Flexstation 3 plate reader (Molecular Devices) using the following settings: λ_ex_ = 335 nm, λ_em_ = 520 and 620 nm, delay 50 μs, integration time 400 μs. Binding was quantified as the ratio of fluorescent signal at 520 nm to that at 620 nm, after subtraction of ratio obtained in the absence of FITC-ligands.

**High content imaging assay**

HEK293-SNAP-GLP-1R cells were seeded into 96-well clear bottom black microplates and labelled with the cleavable SNAP-tag probe BG-SS-649 before inducing internalization by treating cells in serum-free media with agonist at a range of concentrations. Mesna in alkaline TNE buffer (pH 8.6) was applied at the end of the incubation period for 5 min to cleave residual surface GLP-1R. Epifluorescence and transmitted phase contrast images covering ~60% of the well surface were acquired using an automated wide-field microscope with 0.45 NA 10X air objective, and internalised GLP-1R was quantified by fluorescence intensity from cell-containing regions as demarcated by using phase contrast images.

**Pharmacokinetic study**

Animals (*n*=45) were procured from Laboratory Animal Resources Dept. of Sun Pharma Advanced Research Company Ltd. ([www.sparc.life](http://www.sparc.life), a subsidiary of Sun Pharma Industries Ltd.). Animals were acclimatized for one day. On day 0, each animal was weighed. Animals were divided into 9 groups, each group containing 5 male animals (one group representing one time-point). All the animals were injected subcutaneously at a dose of 1 mg/kg (dose volume: 10 ml/kg) using 26½ gauge needle attached to an appropriately graduated syringe. At 1, 2, 4, 8, 12, 24, 48, 72 and 96 hours after injection, ~500 µL of blood was collected from mice by retro-orbital plexus puncture using capillary micro-centrifuge tubes containing anticoagulant (15 µL of 10% K_2_EDTA per tube). Plasma was separated from the collected blood samples by centrifugation at ~3300 rpm (1985 g) for 10 min at 4°C ± 2°C. GL0034 concentrations were estimated using liquid chromatography with tandem mass spectrometry (LCMS-MS). Lower limit of quantitation (LLOQ) for GL0034 was 50 ng/mL.

| **Age (Years)** | **Sex** | **BMI (Kg/m^2^)** | **HbA1c (%)** | **COD** | **Origin** |
| --- | --- | --- | --- | --- | --- |
| 63 | M | 29.4 | - | CVD | Pisa |
| 76 | F | 23.9 | - | CVD | Pisa |
| 32 | M | 24.8 | 4.5 | - | Alberta |
| 47 | M | 40.3 | 5.5 | - | Alberta |
| 64 | F | 22.07 | - | CVD | Pisa |
| 53 | F | 27.2 | - | - | Milan |

**Supplementary Table 1**: Human islet preparations used in the study. COD, cause of death, CVD, cardiovascular disease.

|  | **Glucose AUC 48 hr (mean mmol/L.hr ± SD)** | **Body weight change 48 hr (mean % ± SD)** |
| --- | --- | --- |
| Diabetic control | 1415 ± 113 | 0.9 ± 1.7 |
| Semaglutide 14 nmol/kg | 887 ± 172 * | -0.5 ± 8.9 |
| Semaglutide-Leu32, 7 nmol/kg | 806 ± 185 * | -1.0 ± 1.9 |
| Semaglutide-Leu32, 14 nmol/kg | 677 ± 151 * | -2.8 ± 1.4 |
| Semaglutide-Leu32, 21 nmol/kg | 641 ± 130 *,^#^ | -3.2 ± 2.5 |

**Supplementary Table 2.** Internal screening of C-terminal Leu semaglutide analogue (Semaglutide-Leu32) in *db/db* mice, *n*=8. *p<0.05 *vs.* diabetic control, ^#^p<0.05 *vs.* semaglutide by one-way ANOVA with Bonferroni test.
